# Pancreaticopleural Fistula: Revisited

**DOI:** 10.1155/2012/815476

**Published:** 2012-01-31

**Authors:** Norman Oneil Machado

**Affiliations:** Department of Surgery, Sultan Qaboos University Hospital, Muscat, Oman

## Abstract

Pancreaticopleural fistula is a rare complication of acute and chronic pancreatitis. This usually presents with chest symptoms due to pleural effusion, pleural pseudocyst, or mediastinal pseudocyst. Diagnosis requires a high index of clinical suspicion in patients who develop alcohol-induced pancreatitis and present with pleural effusion which is recurrent or persistent. Analysis of pleural fluid for raised amylase will confirm the diagnosis and investigations like CT. Endoscopic retrograde cholangiopancreaticography (ECRP) or magnetic resonance cholangiopancreaticography (MRCP) may establish the fistulous communication between the pancreas and pleural cavity. The optimal treatment strategy has traditionally been medical management with exocrine suppression with octreotide and ERCP stenting of the fistulous pancreatic duct. Operative therapy considered in the event patient fails to respond to conservative management. There is, however, a lack of clarity regarding the management, and the literature is reviewed here to assess the present view on its pathogenesis, investigations, and management.

## 1. Introduction

Pancreaticopleural fistula has been recognized as a clinical entity since case reports were published in late 1960s [1]. Since that time, pancreaticopleural fistulae and pancreatic ascites have been termed as internal pancreatic fistulae which share common pathogenesis which includes the disruption of main pancreatic duct, resulting in leakage of pancreatic fluid [2–6]. This rare entity may be seen in patients with acute and chronic pancreatitis or may follow traumatic and surgical disruption of the pancreatic duct [2–6]. It is characterized by massive pleural fluid and has a tendency to recur following treatment. While conservative management with pancreatic duct stenting and inhibition of pancreatic secretion with octreotide may achieve closure of fistula in 31 to 45% of cases, surgery leads to healing in 80 to 90% of cases but carries a mortality up to 10% [2–6].

## 2. Incidence

Pleural effusion due to pancreaticopleural fistula is extremely unusual accounting for less than 1% of cases [7]. It is seen in 3 to 7% of patients with pancreatitis [8], and a combined frequency of internal pancreatic fistula (pancreatic ascites and pancreaticopleural fistulae) is seen in between 0.4 and 7% of chronic pancreatitis patients and in 6 and 14% of patients with pseudocyst [5, 6]. Pancreaticopleural fistulae are however more unusual than pancreatic ascites. It usually presents as large recurrent pleural effusion in either pleural space, but left-sided effusion is more common and are reported to account for 76% of cases [2–6].

## 3. Pathophysiology

The development of pancreaticopleural fistula is usually a consequence of leak from an incompletely formed or ruptured pseudocyst [2–6] or in a minority of cases due to direct pancreatic duct leak [4–6]. The fistulous tract passes either through the aortic or oesophageal diaphragmatic orifice or directly transdiaphragmatically. Similar pathophysiology and aetiology apply to pancreatic ascites and pancreatic pleural effusion. If the pancreatic duct disruption occurs anteriorly and is not walled off, a pancreaticoperitoneal fistula will develop that will manifest as ascites [2–6]. If the disruption develops posteriorly, pancreatic secretion will flow into retroperitoneum and may dissect through aortic or oesophageal hiatus into mediastinum and form a pleural fistula or present as mediastinal pseudocyst which in turn ruptures into the pleural cavity and forms a pleural fistula [4–6].

## 4. Clinical Features

Middle-aged men between 40 and 50 years who have history of chronic alcoholism and develop pancreatitis, form the common group of patients who develop pancreaticopleural fistula [3–6]. About half of the patients do not have history of pancreatitis [6]. Trauma is less common cause and is seen in 0.5% of the cases [6]. Pancreatic pseudocyst may be noted in 69 to 77% of the patient, who develop pancreaticopleural fistula [4–6]. The clinical manifestations are often misleading as symptoms are usually associated with significant pleural effusion and consist of dyspnea, cough, chest pain fever and septicaemia [2–8]. Pulmonary symptoms are more common than abdominal symptoms and are usually the presenting symptom with dyspnea being the most common [7, 8]. Rarely do patients complain of abdominal pain typical of acute pancreatitis. The average duration of symptoms is 5.6 weeks [2, 4, 6].

Pleural effusion are predominately left sided; however, right-sided and bilateral effusion occurs in 19% and 14% of patient's, respectively [3]. Pleural effusion of this nature tends to be large and recurrent despite repeated thoracocentesis [2–6]. Many patients go through extensive pulmonary evaluation before pancreas is identified as the site of primary pathology. The pleural effusion is associated with ascites in 20% and pericarditis in 4% [9]. The major complication in these patients is superinfection which contributes to significant morbidity and mortality. In a review of 113 cases from Japan, 98% of the cause of pancreaticopleural fistula was related to chronic relapsing pancreatitis due to chronic alcoholism [10]. Chest pain was the predominant symptom and was noted in 68% with other symptoms being abdominal symptoms and pancreatic ascites in 24% and 12%, respectively [10].

## 5. Diagnosis

Frequently the diagnosis is delayed [2–6, 10]. The time to diagnosis is reported to range from 12 to 49 days [4]. Delay in diagnosis is a critical issue. It needs a high index of clinical suspicion in those with a history of acute pancreatitis and alcohol abuse presenting with a pleural effusion which reforms relatively rapidly after aspiration and for which there is no obvious other cause.

Pleural effusion associated with pancreaticopleural fistula should be distinguished from the small reactive, self-limiting left-sided effusion that commonly occurs in 3 to 17% of patients with acute pancreatitis [3]. A pancreaticopleural fistula may be suspected on the basis of the clinical picture and analysis of pleural fluid following thoracocentesis which reveals an extremely elevated pleural fluid amylase level (normal <150 IU/L), lipase, and high albumin content (>3 g/dL) [2–6]. The serum amylase on the other hand is usually mildly elevated but not invariably and is thought to be partly secondary to reabsorption of amylase from pleural surfaces [4–6]. The differential diagnosis for amylase-rich pleural effusion includes acute pancreatitis, cancer of lung, rectum, breast, female reproductive system, metastatic carcinoma, pneumonia, oesophageal perforation, lymphoma, leukaemia, liver cirrhosis, hydronephrosis, and pulmonary tuberculosis [11].

A simple chest radiography is the first line of investigation, but this provides only limited information of the fluid collection in pleural cavity [3, 4]. A CT scan of the chest and abdomen is valuable in the diagnosis [2–6]. Currently CT is the gold standard for investigating pleural effusion [3, 4]. It is very useful in determining the site and size of effusion, but overall ability to provide accurate delineation of the fistula is disputable [3, 11]. CT abdomen in addition will reveal changes of pancreatitis and identify other associated abnormalities such as pancreatic pseudocysts [2–6]. CT scan may demonstrate the fistulous tract especially if obtained immediately after an ERCP. Magnetic resonance cholangiopancreaticography is reported to be particularly useful in demonstrating the pancreatic pathology and the fistula [5, 8]. It is a noninvasive alternative to ERCP, visualizes the duct beyond the strictures, depicts parenchymal atrophy, ductal anatomy and small intrapancreatic and extrapancreatic pseudocyst, peripancreatic collection, or pancreaticopleural fistula, and is useful where ERCP fails to give adequate information [5, 8].

The diagnosis may be confirmed with ERCP although it may not always be possible to demonstrate the fistulous tract [6]. ERCP may not demonstrate the fistula in patients in whom the site of ductal disruption exists in more distal side than the site where a ductal obstruction exists. In these cases, CT or MRCP may be helpful. ERCP leads to diagnosis in 80% of cases and demonstrates the fistulous tract in 59% to 74% of the cases [3, 4, 11]. Visualisation of the entire pancreatic ductal tree is useful in planning a rational surgical approach, particularly in deciding between a resection and a drainage procedure [2, 6].

## 6. Treatment

Due to high failure rate in the past of simple conservative management including drainage of chest and keeping the patient nil per oral, the patient would invariably require surgical intervention. Following the encouraging results with octreotide administration and stent placement, success rate of conservative management has significantly improved [2–7, 10, 11]. The available treatment modalities now include (1) conservative/medical management (octreotide and thoracentesis), (2) ERCP plus/minus endoscopic pancreatic stent placement, and (3) surgery [2–7, 10, 11].

The aim of the medical treatment is to reduce stimulation to pancreatic exocrine secretions [2, 4, 6, 10, 11]. Medical treatment constitutes thoracocentesis and/or tube thoracostomy, both of which encourage apposition of serosal surfaces and symptom relief along with the administration of somatostatin analogues [4, 11]. The duration of this treatment by itself varies, but in the past when endoscopic stenting was not available, conservative management did not exceed 2 to 4 weeks [6, 9]. Within this time if there was failure to respond to conservative management, which includes failure of pleural effusion and or superinfection to clear, then surgical intervention would be the optimal treatment. Presently, due to the success achieved with octreotide administration and placement of stent in the duct, longer period of conservative management is employed; this includes octreotide being continued from 2.5 to 6 months and chest being drained from 6 to 24 days [4]. Octreotide is given as an initial dose of 50 ug, administered subcutaneously three times a day, and the dose is titrated based upon the fistula output, the maximal dose employed being 250 ug three times daily [4, 11]. It is reported that octreotide significantly reduces fistula output and decreases the time to fistula closure [11]. Measures like the prohibition of oral intake, nasogastric tube insertion and total parenteral nutrition used in the past are no longer necessary [4–6, 11]. Complications related to nonoperative therapy including malnutrition, central venous catheter infections, deep vein thrombosis, and sepsis associated with intestinal mucosal atrophy from prolonged fasting have been significantly reduced following the use of octreotide and pancreatic stent placement [6, 12]. Moreover, substantial overall morbidity and cost accrued due to prolonged hospitalization are reduced [4, 6, 12].

ERCP and stent placement have revolutionized the concept of nonsurgical management in these patients [2–7]. The potential benefits of ERCP include papillary sphincterotomy in cases of sphincter of Oddi dysfunction, dilatation of stenosis of the main pancreatic duct, and extraction of stones from the main pancreatic duct with or without extracorporeal lithotripsy, all of which could contribute to persistence of the fistula [9, 11, 13, 14]. Mere endoscopic papillotomy may precede the insertion of the stent as a lesser option in those cases where stenting fails [4]. When stents are placed into the main pancreatic duct, patient's pain caused by ductal pressure may be relieved; moreover, pseudocysts that communicate with the main pancreatic duct may be drained [4–7, 13–15]. ERCP also facilitates drainage of fistula by the insertion of nasopancreatic drains for 1 week, followed by placement of an endoprosthesis in the pancreatic duct [6, 13–15]. The distinct advantage of naso pancreatic drain in contrast to stent placement is that it allows pancreaticograms to be obtained repeatedly without further invasive procedures. It also allows application of low intermittent suction, which may potentially facilitate closure of a leak or fistula [15]. However, the major drawbacks include the necessity for continued hospitalisation and patient discomfort due to the presence of the tube in the nose [15]. ERCP in general is an invasive procedure, and the availability of expertise in endoscopic placement of stent is a bare necessity [6, 11].

The main objective of stent placement other than decompressing the duct is to bridge the site of duct disruption if possible [6, 13–15]. Most fistulae appear to arise from head or body of the pancreas and are thus amenable to bridging with a pancreatic stent [6, 13, 15]. However bridging may not be feasible in patients in whom the fistula arises from the tail of the pancreas and the stent may have to be placed close to the duct disruption [6, 13–15]. Bridging pancreatic stents helps to close the fistula rapidly by decreasing the ductal pressure and abolition of pancreatic pressure gradient, achieved by bypassing the sphincter of Oddi and stricture and by mechanically blocking the fistula lumen. The stents used for this purpose are either 5 Fr or 7 Fr size [6, 13–15]. Fistulae from a pseudocyst which is no longer in direct communication with pancreatic ductal system may heal spontaneously [6, 15].

The principal aim of the treatment with stent is to achieve drainage of ducts with fistulae in short term and drainage of the stenosed pancreatic duct in long term (2–12 months) [15]. The optimum duration of drainage for fistulae is unknown at present. This can vary from 4 to 12 weeks [6, 9]. One approach would be to assess the persistence of fistula by repeating ERCP at 6 weekly intervals and documenting the passage of dye into the chest [4]. The concern, however, of long-term use of stent is that it itself causes ductal changes that do not always regress after its removal [16]. As the data on the long-term consequence of pancreatic duct stent placement is lacking, a definite opinion is difficult to draw [6, 12]. Significant proportion of these patients, however, may still require surgery particularly for persistent, recurrent fluid collections secondary to stenosis or disruption of the main pancreatic duct [17]. The issue of how long to continue with endoscopic treatment is largely unresolved [4].

**Figure 1:**
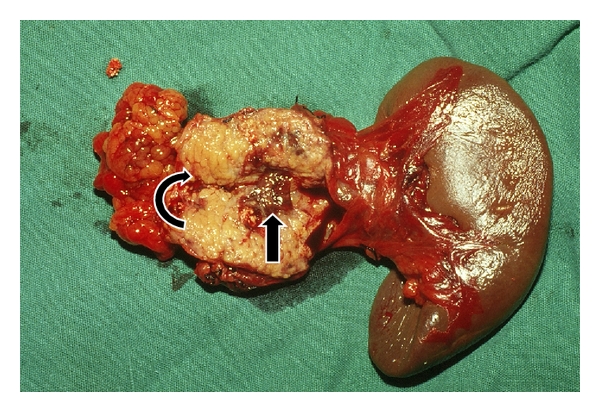
Distal pancreatectomy specimen of a patient with pancreaticopleural fistula in chronic pancreatitis who failed to respond to conservative management. Pancreatic duct with strictured segment (curved arrow) and site of leak (straight arrow) seen.

Surgical treatment is safe and effective and is appropriate either when medical management fails or where underlying condition requires surgical intervention [2–7, 10, 11]. The main indications for surgery are failure of conservative and endoscopic treatment, obstruction of pancreatic duct that cannot be managed endoscopically and a symptomatic fit patient [2–7, 10, 11]. Surgical treatment includes either some form of a pancreatic resection (Figure 1) or enteropancreatic anastomosis to the site of pancreatic duct leakage or to the pseudocyst [4, 7, 11]. If there is an obstruction of the main pancreatic duct proximal to the fistula, surgical treatment is necessary to decompress the obstructed duct with or without excision of the involved portion of the obstructed pancreas [2–6, 10, 11]. Cystogastrostomy, cystojejunostomy and distal and middle pancreatectomy are appropriate options in the setting of symptomatic pancreatic pseudocysts or pancreatic duct obstruction [2–6, 11] (Figure 1).

In summary the first line of treatment should be chest drain, octreotide therapy, and ERCP with an attempt at pancreatic stent insertion. Endoscopic pancreatic stenting is effective therapeutic option associated with minimal morbidity and mortality, and combined with somatostatin analogues it can shorten the duration of hospital stay. Surgical intervention is the 2nd line of treatment with an appreciable morbidity and mortality.

 King et al. based on the review of 63 adult patients in the literature between 1970 and 2008 made the following observation [2]. The majority of the patients were male (71%), and there was predominance of alcohol-induced chronic pancreatitis (51%). Complications were noted in 16% of patients and death in 3%. Most patients were treated initially with medical therapy (87%). Medical therapy was deemed to have failed after an average period of  35 ± 5  days. Total duration of therapy for patients in whom operative intervention was required after attempted medical management was  40 ± 6  days which was greater than the surgery alone cohort [2]. Based on this review, they concluded that the traditional strategy of initial medical management followed by operative treatment is questionable. Medical therapy was found to be successful only in 31% of the time whereas operation therapy was more than 3 times likely to succeed (94%) when applied as either an initial strategy or after failed medical management [2].

Analyzing the advantages of early surgical intervention based on the review, they felt that failed initial attempts at medical therapy would lead to longer periods of treatment than in those who underwent operative treatment early on. Moreover, the time spent on treating patients medically was 50% more than that was devoted to postoperative recovery, indicating that earlier operative intervention may have decreased the duration of therapy required for resolution of fistula by 50% [2]. Further advantage noted was shorter postoperative recovery time for patients experiencing morbidity (16 ± 3 versus  24 ± 8  days) which may reflect the widespread trend toward shorter postoperative hospital stay in recent years [2]. It was also noted that 70% of the complications that followed surgical intervention was seen in patients in whom conversion from medical to operative therapy was necessary. These complications included leaks, recurrence of fistula, intra-abdominal infection, wound infection, and the development of diabetes. In general, conservative treatment for pancreaticopleural fistula has a success rate of 30–60%, with a recurrence rate of 15% and mortality of 12%; in contrast, operative therapy has a success rate of 90% with up to 18% recurrence rate [2].

## 7. Conclusion

Pancreaticopleural fistula is difficult to diagnose and at times difficult to treat. They require a high index of clinical suspicion to diagnose, particularly in the setting of recurrent pleural effusions with coexisting history of pancreatitis or alcohol abuse. The predominant symptoms are related to chest rather than abdomen. Early pleural fluid amylase testing will avoid delayed diagnosis. The initial line of treatment includes drainage of the effusion, the inhibition of pancreatic secretions with octreotide and possibly ERCP plus stenting of the pancreatic duct. While surgery is generally considered to be appropriate when medical measures fail or if there is an associated or complicated pseudocyst, there are others who support an early surgical intervention to reduce the recovery time and increase the success rate.
